# Complexity of the Immune Response Elicited by Different COVID-19 Vaccines, in the Light of Natural Autoantibodies and Immunomodulatory Therapies

**DOI:** 10.3390/ijms24076439

**Published:** 2023-03-29

**Authors:** Katalin Böröcz, Ágnes Kinyó, Diana Simon, Szabina Erdő-Bonyár, Péter Németh, Timea Berki

**Affiliations:** 1 Department of Immunology and Biotechnology, Clinical Center, University of Pécs Medical School, 7624 Pécs, Hungary; 2 Department of Dermatology, Venereology and Oncodermatology, Clinical Center, University of Pécs Medical School, 7624 Pécs, Hungary

**Keywords:** COVID-19, vaccine, natural autoantibodies, ELISA, immunomodulation, humoral, cellular response

## Abstract

Despite the abundance of data on the COVID-19 vaccine-induced immune activation, the impact of natural autoantibodies (nAAbs) on these processes is less well defined. Therefore, we investigated potential connections between vaccine efficacy and nAAb levels. We were also interested in the impact of immunomodulatory therapies on vaccine efficacy. Clinical residual samples were used for the assessment of the COVID-19 vaccine-elicited immune response (IR) (n=255), as well as for the investigation of the immunization-associated expansion of the nAAb pool (n=185). In order to study the potential interaction between immunomodulatory therapies and the vaccine-induced IR, untreated, healthy individuals and patients receiving anti-TNFα or anti-IL-17 therapies were compared (n total =45). In-house ELISAs (anticitrate synthase, anti-HSP60 and-70) and commercial ELISAs (anti-SARS-CoV-2 ELISAs IgG, IgA, NeutraLISA and IFN-γ release assay ‘IGRA’) were applied. We found significant differences in the IR given to different vaccines. Moreover, nAAb levels showed plasticity in response to anti-COVID-19 immunization. We conclude that our findings may support the theorem about the non-specific beneficial ‘side effects’ of vaccination, including the broadening of the nAAb repertoire. Considering immunomodulation, we suggest that anti-TNFα and anti-IL17 treatments may interfere negatively with MALT-associated IR, manifested as decreased IgA titers; however, the modest sample numbers of the herein presented model might be a limiting factor of reaching a more comprehensive conclusion.

## 1. Introduction

### 1.1. COVID-19 Vaccines and Immunoserology

Since the last days of 2020 (and the initial days of 2021), four different anti-SARS-CoV-2 vaccines have been most frequently used to immunize the Hungarian population: the Pfizer/BioNTech vaccine using lipid nanoparticles to deliver mRNA, the adenovirus-based Oxford–AstraZeneca COVID-19 and Sputnik V vaccines, and the Sinopharm BBIBP-CorV (Vero cell) vaccine that contains the whole inactivated virus [1]. Using serologic assays, we evaluated and compared the immune responses triggered by the different vaccines.

### 1.2. Natural (Auto)Antibodies (nAAbs)

Natural antibodies (nAbs) are produced by B1, follicular and marginal zone (MZ) B cells [2]. Natural antibody-producing B-1 B cells are considered an intermediate stage of evolution between innate and adaptive immunity [3]. Circa 5% of the B cell population are considered to be B1 cells, amounting to 5 × 10^7^ B1 cells in an average human, which suggests that nAbs are a significant part of the systemic antibody pool [4]. 

Although nAbs are known for their broad reactivity against pathogens and self-antigens, some have the ability to recognize evolutionarily fixed epitopes present in foreign antige. Of these latter ones, the most well-characterized epitopes include phospholipids, oxidized lipids, glycolipids and glycoproteins [5].

Natural antibodies are involved in the pathogenesis of autoimmunity, but only a minority of nAbs and nAAbs have pathogenic features [4]. Many individuals possess antibodies directed against common epitopes in highly mutating viral infections, such as influenza and HIV. These so-called “broadly neutralizing antibodies” share some characteristics with nAbs [4].

The traditional definition states that nAbs are present prior to antigen stimulation, providing a first line of defense against infection [5]. Importantly, nAbs provide various essential functions within the immune system, ranging from scavenger activity by elimination of apoptotic cells and cell debris to the ability to provide protection against bacterial, viral and fungal infections [5].

Indeed, nowadays it is supposed that the nAb repertoire is inherently linked to the host biome [6,7]. This explains how vaccination—one of the main pillars of modern medicine—induces not only the formation of memory B cells and antibodies that confer immunity to disease-causing pathogens, but also has an unintended impact on the natural antibody repertoire [7,8].

The dogma that high-affinity IgG response is the major goal of immunization and low-affinity nAbs should be avoided has positively contributed to the lack of information regarding the role of nAbs in vaccination [9]. However, it has been recently proposed that nAbs may serve as potential screening targets to predict the strength of the antigen-induced immune response [10]. 

### 1.3. Citrate Synthase (CS) as a Natural Autoantibody 

The mitochondrial citrate synthase (CS) enzyme exists in nearly all living cells and is a key enzyme in the first step of the citric acid cycle. It is one of the most conserved proteins both structurally and functionally. CS is a quantitative enzyme marker for the presence of intact mitochondria. Naturally occurring autoantibodies against different epitopes of CS—as a part of the network of natural autoantibodies—are present in healthy individuals and show dynamic changes in chronic diseases of immune origin [3,11,12,13,14].

### 1.4. Nonspecific Effects of Vaccines (NSEs)

Nonspecific effects of vaccines are often dismissed or ignored because they are difficult to explain biologically [15]. It has already been described in cases of laboratory rats that immunization enhances the natural antibody repertoire. In other words, immunization is associated with a larger natural antibody repertoire and stronger antibody binding, which also implies an enhanced total nAb binding ability [7]. Earlier studies also described that the measles vaccine (MV) had beneficial nonspecific side effects (NSEs) [16,17,18]. In a review of the potential NSEs on child mortality of the BCG, DTP and MV, this latter vaccine was associated with a 46% reduction in mortality [18]. The measles, mumps and rubella (MMR) vaccine, used in high-income countries (e.g., Denmark and the USA) instead of MV, has also been associated with beneficial NSEs [19,20]. BCG vaccine studies examining in vitro IFN-γ responses and measles vaccine studies examining lymphoproliferation to microbial antigen stimulation consistently suggest nonspecific immunological effects [21].

Herein, we would like to suggest that a better understanding of the link between the adaptive and natural arms of immunity may support the theorem that pathogen-associated stimulation of the immune system leads not only to specific immunological memory (represented by memory T and B cells) but also to changes in the nAb repertoire. This type of plasticity may also result in long-term immune modulatory effects [15]. In order to achieve more efficient vaccination practices, we need to understand how training of the immune system can be optimized to increase general disease resistance and decrease vaccine failure. Likewise, we need to understand which individual parameters may be screened to predict vaccine efficacy [17].

### 1.5. Heat Shock Protein 60 and 70 (HSP60 and HSP70)

Autoantibody profiling is important to gain a better understanding of the biomarkers used by the immune system. For instance, heat shock HSP60 and HSP70 are prominent examples of immune biomarker molecules of underrated importance [22]. It has been recently described that human cord blood contains IgM autoantibodies to various peptide epitopes of both HSP60 and HSP70 [22]. Based on this finding, it can be hypothesized that natural autoantibodies to HSP60 and HSP70 seem to be part of the healthy immune system [22]. HSPs are ubiquitously found in almost all living organisms studied so far. The DNA sequence that makes up this family of genes is highly conserved across species. This family of genes was named because of their expression after exposure to heat [23]. 

However, the genes are now known to be induced by a wide variety of environmental or metabolic stresses that include the following: anoxia, ischemia, heavy metal ions, ethanol, nicotine, surgical stress and viral agents (11). Most organisms, including viruses such as coronavirus, require HSPs to protect the cellular system and balance its proteomic system. In short, HSPs act as housekeepers in the cellular system of the viruses for them to acclimatize to the host (i.e., to a new environment) [23,24,25,26,27,28]. Focusing on the direct connections between SARS-CoV-2 and HSPs, it has been recently described that HSP60 has been implicated in inappropriate inflammatory reactions that exacerbate the progression of COVID-19 [29,30], while two of the HSP70s in host cells have been implicated in the modulation of SARS-CoV-2. Hsp70A1L was reported to be epigenetically modulated by SARS-CoV-2, among other genes, including those from the Hsp70A1L family of proteins [31,32].

### 1.6. COVID-19 and Autoimmunity 

Vaccine-induced autoimmunity is a known phenomenon [33,34,35]. A link between COVID-19 and the development of autoimmunity has been reported [36,37]. A possible explanation could be molecular mimicry between the virus and human proteins, where immune responses raised against SARS-CoV-2 cross-react with human proteins that share peptide sequence molecular shapes with the virus. Peptide sharing has been found between SARS-CoV-2 antigenic epitopes and different human proteins, including chaperones [36,37,38].

### 1.7. Serologic Response to COVID-19 Vaccines in Patients Treated with Biological Therapies

It has already been a matter of concern that biologics may increase infection risks [39,40]. 

It has long been known that in the pathogenesis of psoriasis, a self-sustaining cycle of inflammation plays an important role. This is mediated mainly by T cells and cytokines such as TNFα, IL-23 and IL-17 [41,42]. Biological agents targeting these cytokines are key players in the treatment of psoriasis [42]. Anti-TNFα and anti-IL-17 are prominent examples for different classes of biologics that are used to treat moderate-to-severe psoriasis and hidradenitis suppurativa [43]. 

The biological therapy using the human IgG1κ monoclonal antibody (mAb) works on the principle that the administered mAb binds to the protein interleukin (IL)-17A, thus exerting an inhibitory function. It may be prescribed for the indication of moderate-to-severe plaque psoriasis, active enthesitis-related arthritis, active psoriatic arthritis, ankylosing spondylitis, active nonradiographic axial spondyloarthritis and objective signs of inflammation. The biological drug inhibiting tumor necrosis factor (TNFα) is an important treatment in a number of inflammatory conditions, including rheumatoid arthritis (RA), spondyloarthritis, psoriasis, hidradenitis suppurativa and inflammatory bowel disease (IBD) [44,45].

Theoretical data from previous coronavirus outbreaks have suggested a strong role for type I interferon, B cell-released antibodies, tumor necrosis factor and other cytokines in the viral immune response [39]. Interleukin (IL) 17 cytokines are important for immune cell recruitment to infection sites to promote clearance, while also activating downstream cascades of cytokines and chemokines [39]. For these reasons, the question has emerged of whether stopping immunomodulatory therapy can reduce the infection-associated risks during the COVID-19 outbreak [40]. For this subject, divided opinions are found in the literature. It has been described that blocking TNF signaling may save lives in the COVID-19 pandemic [46] and that discontinuation of biological treatments can result in the loss of response when treatments are reintroduced or even result in the formation of antibodies to the discontinued biologic [40]. 

Anti-TNFα and IL-17 are drugs supplied with warnings about an increased risk of infections [44,47,48]. It has already been described that patients treated with anti-TNFα exhibited lower serologic responses one month and six months after vaccination (COVID-19 BNT162b2) compared to those not treated with anti-TNFα or to healthy controls (HCs) [44]. Additionally, compared with the placebo group, anti-TNFα-treated patients showed a moderate increase in susceptibility to upper respiratory infections (URIs) [49]. Though serum levels of TNFα have been noted to be elevated in patients with viral respiratory illnesses such as influenza, no significant elevation of TNFα was detected in the serum of patients with SARS, suggesting that the immune response to SARS may differ from that of other respiratory illnesses [42]. 

Contradictorily, considering anti-IL-17, it has also been described that the risk of serious infections is unchanged or low over the short term; therefore, in an acute setting of COVID-19, the benefit of anti-IL-17 might outweigh the risks of the infection [49]. The latest common position regarding IL-17 inhibition is that it does not interfere significantly with the capacity of patients to develop the expected responses to SARS-CoV-2 [42]. 

## 2. Results

### 2.1. Anti-SARS-CoV-2 Vaccination- or Infection-Induced IgG and IgA Antibody Levels

Between the unvaccinated, uninfected controls and all the other groups, significant differences were found in all cases (*p* < 0.001); markers are not shown on the figure.

According to Table 1, the highest sero-positivity ratios in terms of anti-SARS-CoV-2 IgG were measured in the mRNA and the vector vaccine groups, while the highest sero-positivity ratios in terms of anti-SARS-CoV-2 IgA were detected in the mRNA and the unvaccinated, infected groups.

**Figure 1:**
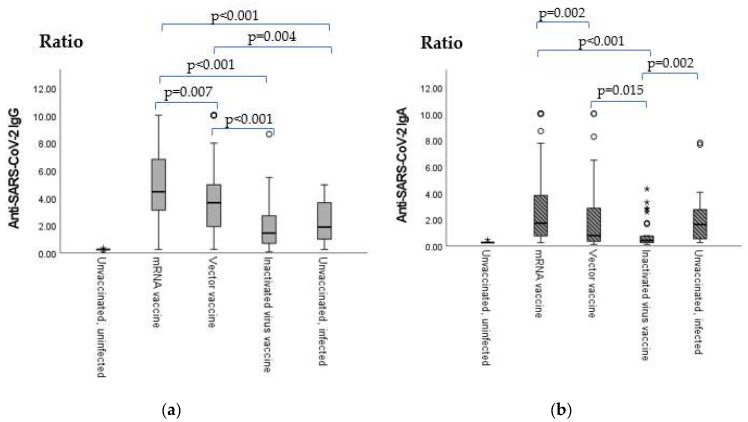
Anti-SARS-CoV-2 IgA and IgG antibody titer results are expressed as a ratio of the extinction of the patient sample (or control) over the extinction of the calibrator. Ratio < 0.8—negative; ratio ≥ 0.8 to 1.1—borderline; and ratio > 1.1—positive. (**a**) Vaccination group-based comparison of anti-SARS-CoV-2 IgG titers. (**b**) Vaccination group-based comparison of anti-SARS-CoV-2 IgA titers. Sample numbers: mRNA vaccine (Pfizer–BioNtech) = 106, vector vaccine (AstraZeneca and Sputnik V) = 77, inactivated virus vaccine (Sinopharm) = 34, unvaccinated, uninfected individuals (used as the negative control) = 9, and unvaccinated, infected individuals (used as the positive control) = 27. Total = 255.The sample numbers were as follows: mRNA vaccine (Pfizer–BioNtech) = 106, vector vaccine (AstraZeneca and Sputnik V) = 77, inactivated virus vaccine (Sinopharm) = 34, unvaccinated, uninfected individuals (used as the negative control) = 9, and unvaccinated, infected individuals (used as the positive control) = 27. Total = 255.

In terms of anti-SARS-CoV-2 IgG titers, statistically significant differences were found between the inactivated virus vaccine group and the mRNA group, as well as between the inactivated virus vaccine group and the vector vaccine group (*p* < 0.001 in both cases). Vaccinees of the mRNA and the vector vaccine groups also showed significantly higher IgG antibody titers than the unvaccinated, infected individuals (*p* < 0.001 and *p* = 0.004, respectively). The mRNA vaccines elicited significantly higher antibody titers than the vector vaccines (*p* = 0.004) (Figure 1a).

In terms of anti-SARS-CoV-2 IgA titers, the vector vaccine and the unvaccinated, infected groups as well as the mRNA vaccine group showed significantly higher antibody levels than the inactivated virus vaccine recipients (*p* = 0.0015, *p* = 0.002 and *p* < 0.001, respectively). Vaccinees immunized with the mRNA vaccine mounted significantly higher SARS-CoV-2 IgA titers than vaccinees of the vector vaccine group (*p* = 0.002) (Figure 1b).

### 2.2. Anti-SARS-CoV-2 Neutralizing Antibody Titers and Anti-IFN-γ (IGRA) Levels

In the case of the IFN-γ release assay (IGRA) T cell-mediated IFN-γ release after stimulation with SARS-CoV-2, spike peptides were assessed. In the IGRA test, the calibrator material was adjusted to the international reference material ‘Non WHO Reference Material Interferon Gamma (HUMAN, rDNA derived) NIBSC code: 87/586’. Results are expressed in international units per milliliter (mIU/mL). NeutraLisa test results are given as percentages of inhibition (%IH). In the test kit, the ‘First WHO International Standard for anti-SARS-CoV-2 immunoglobulin (human) NIBSC code: 20/136’ is used as the reference material (for detailed test descriptions, please refer to Section 4).

Between the unvaccinated, uninfected controls and all the other groups, significant differences were found in all cases (*p* < 0.001); markers are not shown on the figure.

According to Table 2, the highest sero-positivity ratios in terms of anti-IFN-γ (IGRA) IgG were found in the mRNA vaccine and the unvaccinated, infected groups, while the highest sero-positivity ratios in terms of the neutralizing antibody titers (NeutraLisa IgA/G/M) were measured in the vector vaccine and the mRNA vaccine groups.

**Figure 2:**
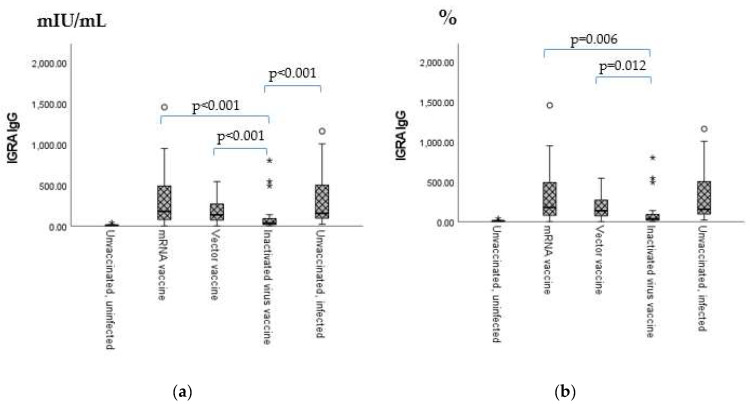
(**a**) Vaccination group-based comparison of antihuman IFN-γ release assay (IgG). (**b**) Vaccination group-based comparison of neutralizing antibody titers (IgA/G/M). The sample numbers were as follows: mRNA-based vaccine (Pfizer–BioNtech) = 106, vector vaccine (AstraZeneca and Sputnik V) = 77, inactivated virus based vaccine (Sinopharm) = 34, unvaccinated, uninfected individuals (used as the negative control) = 9, and unvaccinated, infected individuals (used as the positive control) = 27. Total = 255. The sample numbers were as follows: mRNA-based vaccine (Pfizer–BioNtech) = 106, vector vaccine (AstraZeneca and Sputnik V) = 77, inactivated virus based vaccine (Sinopharm) = 34, unvaccinated, uninfected individuals (used as the negative control) = 9, and unvaccinated, infected individuals (used as the positive control) = 27. Total = 255.

As shown in Figure 2, in terms of anti-IFN-γ (IGRA) titers, there was a significant difference between the inactivated virus vaccine and all the other three vaccination groups (mRNA vaccine, vector vaccine and unvaccinated, infected individuals) (*p* < 0.001 in all cases) (Figure 2a).

In terms of NeutraLisa IgA/G/M titers, significant differences were found between the mRNA vaccine and the inactivated virus vaccine groups (*p* = 0.006), as well as between the vector vaccine and the inactivated virus vaccine groups (*p* = 0.012).

### 2.3. Increase in Antibody Titers of Biological Therapy-Treated Patients from the 4th to 8th Week Post-Vaccination 

**Figure 3:**
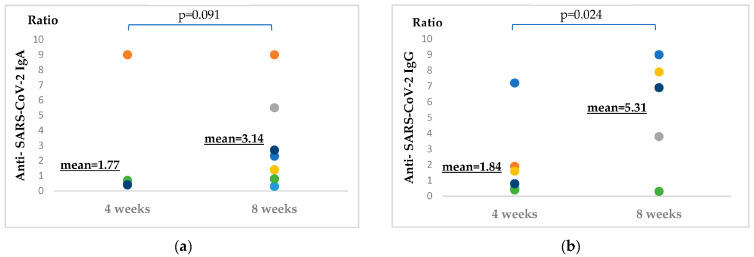
(**a**) Comparison of means of IgA titers. (**b**) Comparison of means of IgG titers. The antibody levels of patients treated with immunomodulatory therapies at weeks 4 and 8 post-immunization were compared, as shown above. Colored dots represent singular patient results. Anti-SARS-CoV-2 IgA and IgG antibody levels of the same set of patients (*n* = 7) were measured before vaccination (used as a negative control, with patients showing no antibodies; data is not shown), and 4 and 8 weeks post-immunization. The population means of the two time points were compared using the paired t-test. Patients had mixed vaccination backgrounds and received anti-TNFα or anti-IL17 therapies, as shown in Supplementary Table S1.

As shown in Figure 3, the means of results of patients with different immunomodulatory therapies (anti-TNFα or anti-IL17) and with mixed vaccination backgrounds (Supplementary Table S1) have been compared to each other, in terms of anti-SARS-CoV-2 IgA and IgG antibody titers. For the sample measurement and subsequent data analysis, three venipunctures have been performed: time point zero was used as a negative control (on the day of immunization and before vaccine administration, when there were no detectable antibody levels), followed by time point one (4 weeks post-vaccination) and time point two (8 weeks post-vaccination). (For the vaccine group nomenclature used for the figures, please see Supplementary Table S2)

The compared time points were the 4th and the 8th weeks post-vaccination. Considering anti-SARS-CoV-2 IgA titers, although the results proved to be visibly higher after 8 weeks, in statistical terms only a trend was detectable (*p* = 0.091). Considering anti-SARS-CoV-2 IgG titers, by the 8th week post-immunization the increase in humoral response was also manifested in statistical terms (*p* = 0.024).

Anti-SARS-CoV-2 IgA and IgG antibody titer results were expressed as a ratio of the extinction of the patient sample (or control) over the extinction of the calibrator. Ratio < 0.8—negative; ratio ≥ 0.8 to 1.1—borderline; and ratio > 1.1—positive.

Sample number = 7 (Supplementary Table S1).

### 2.4. Comparison of Vaccine Efficacy Results of Recipients of Immunomodulatory Therapies with Untreated, Healthy Individuals

**Figure 4:**
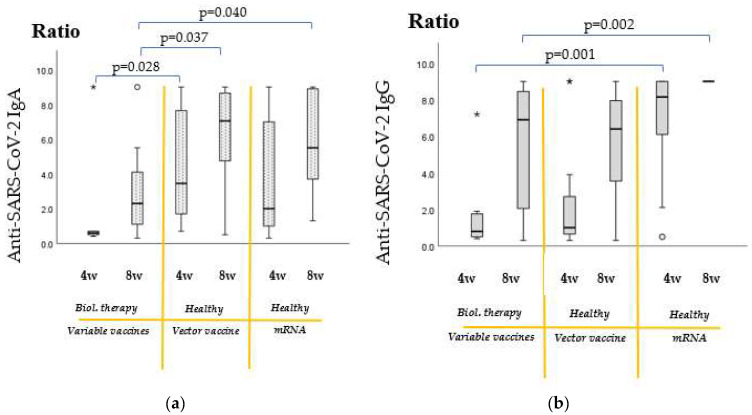
(**a**) Comparison of anti-SARS-CoV-2 IgA titers of recipients of immunomodulatory therapies with untreated individuals. (**b**) Comparison of anti-SARS-CoV-2 IgG titers of recipients of immunomodulatory therapies with untreated individuals.Anti-SARS-CoV-2 IgA and IgG antibody titer results were expressed as a ratio of the extinction of the patient sample (or control) over the extinction of the calibrator. Ratio < 0.8—negative; ratio ≥ 0.8 to 1.1—borderline; and ratio > 1.1—positive. Patients of variable vaccination backgrounds receiving biological therapies (Supplementary Table S1) were compared with healthy, untreated recipients of vector vaccines and healthy, untreated recipients of mRNA vaccines. They were compared at 4 and 8 weeks post-vaccination. For the sample measurement and subsequent data analysis, three venipunctures have been performed: time point zero was used as a negative control (on the day of immunization and before vaccine administration, when there were no detectable antibody levels; data is not shown), followed by time point one (4 weeks post-vaccination) and time point two (8 weeks post-vaccination). (For vaccine group nomenclature used for the figures, please see Supplementary Table S2.)The sample numbers were as follows: untreated, healthy individuals and recipients of the mRNA vaccine (Moderna and Pfizer–BioNtech) = 22. Untreated, healthy individuals and recipients of vector vaccines (AstraZeneca and Sputnik V) = 16. Biological therapy-treated patients with mixed vaccination backgrounds (Pfizer–BioNtech, Moderna, Sputnik V, AstraZeneca and Sinopharm) = 7.

In terms of anti-SARS-CoV-2 IgA levels (Figure 4a), by the 4th week post-immunization, statistically significant differences were detectable between patients receiving biological therapies and healthy recipients of vector vaccines (*p* = 0.028). Interestingly, at this time point, between patients receiving biological therapies and healthy recipients of mRNA vaccines only a tendentious statistical difference (trend) was observable (*p* = 0.088). By the 8th week post-immunization, statistically significant differences were detectable between patients receiving biological therapies and healthy recipients of vector vaccines (*p* = 0.037), as well as between patients receiving biological therapies and healthy recipients of mRNA vaccines (*p* = 0.040). Neither by the 4th nor the 8th week post-immunization were significant statistical differences between untreated vector and untreated mRNA vaccine recipients observed.

In terms of anti-SARS-CoV-2 IgG levels (Figure 4b), by the 4th week post-immunization, statistically significant differences were detectable between patients receiving biological therapies and healthy recipients of mRNA vaccines (*p* = 0.001). At this time point, between patients on biological therapies and untreated recipients of vector vaccines, no statistical difference was observable. By the 8th week post-immunization, statistically significant differences were detectable between patients receiving biological therapies and untreated recipients of mRNA vaccines (*p* = 0.002). Comparing patients receiving biological therapies with untreated recipients of vector vaccines, we can say that by the 8th week post-immunization, their IgG antibody titers reached similarly high levels. Interestingly, by this latter time point, the difference between healthy recipients of vector and mRNA vaccines became significant (*p* < 0.001). 

### 2.5. Dynamic Adaptation of IgM Isotype Anti-CS Natural Autoantibodies (nAAbs)

**Figure 5:**
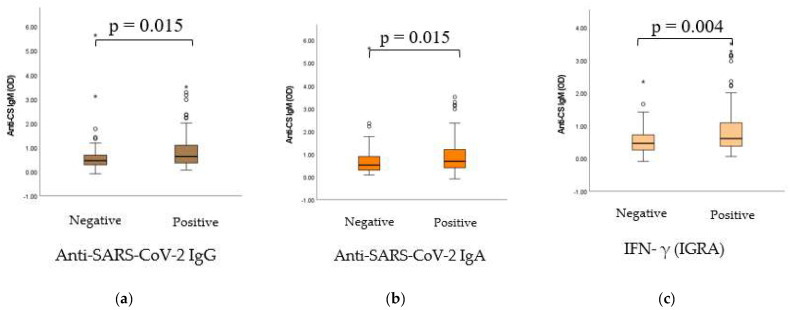
(**a**) Connection between the nAAb CS IgM anti-SARS-CoV-2 IgG positivity. (**b**) Connection between the nAAb CS IgM anti-SARS-CoV-2 IgA positivity. (**c**) Connection between the nAAb CS IgM IGRA (cellular response) positivity. Anti-SARS-CoV-2 IgG, IgA and IFN-γ qualitative (positive and negative) results were evaluated as per the manufacturer’s instructions. Anti-CS IgM absorbance (OD) results were handled as continuous, non-normally distributed integers and the alterations of titers were considered. Data are relative to the undivided, total sample cohort, without distinction between vaccination groups. The sample numbers were as follows: (**a**) anti-SARS-CoV-2 IgG negative/positive = 44/187, (**b**) anti-SARS-CoV-2 IgA negative/positive = 117/123 and (**c**) IFN- γ (IGRA) negative/positive = 53/199. Total = 255.

As shown in Figure 5, significant positive statistical connection was found between the nAAb anti-CS IgM and the immunization-induced humoral and cellular immune response. In the case of positive anti-SARS-CoV-2 IgG, IgA and interferon-γ results, significantly higher anti-CS IgM levels were measured.

### 2.6. Dynamic Adaptation of IgG Isotype Anti-CS Natural Autoantibodies (nAAbs)

**Figure 6:**
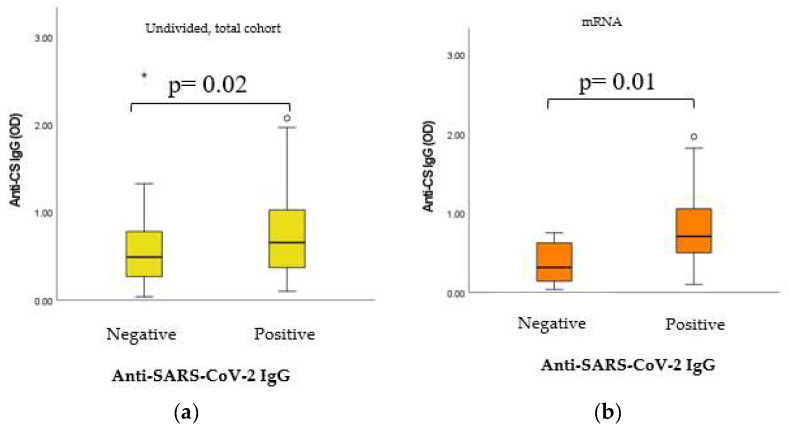
(**a**) Connection between CS IgG titers and anti-SARS-CoV-2 IgG results in the undivided, total sample cohort. (**b**) Connection between CS IgG titers and anti-SARS-CoV-2 IgG results in the mRNA vaccine group. Anti-SARS-CoV-2 IgG qualitative results (positive and negative) were evaluated as per the manufacturer’s instructions. Anti-CS IgG absorbance (OD) results were handled as continuous, non-normally distributed integers and the alterations of titers were considered. In the undivided total cohort, as well as in the mRNA vaccine group, statistically significant positive connection was found between anticitrate synthase IgG levels and the post-immunization anti-SARS-CoV-2 IgG qualitative (positive and negative) results (*p* = 0.02 and *p* = 0.01, respectively). The sample numbers were as follows: (**a**) anti-SARS-CoV-2 IgG negative/positive = 45/132 and (**b**) anti-SARS-CoV-2 IgA negative/positive = 6/62. Total =185.

As shown in Figure 6, significant positive statistical connection was found between the nAAb anti-CS IgG and the immunization-induced anti-SARS-CoV-2 IgG results, both in the case of the undivided, total sample cohort and in the case of the mRNA vaccine group.

### 2.7. Dynamic Adaptation of Anti-HSP70 IgG Antibodies

**Figure 7:**
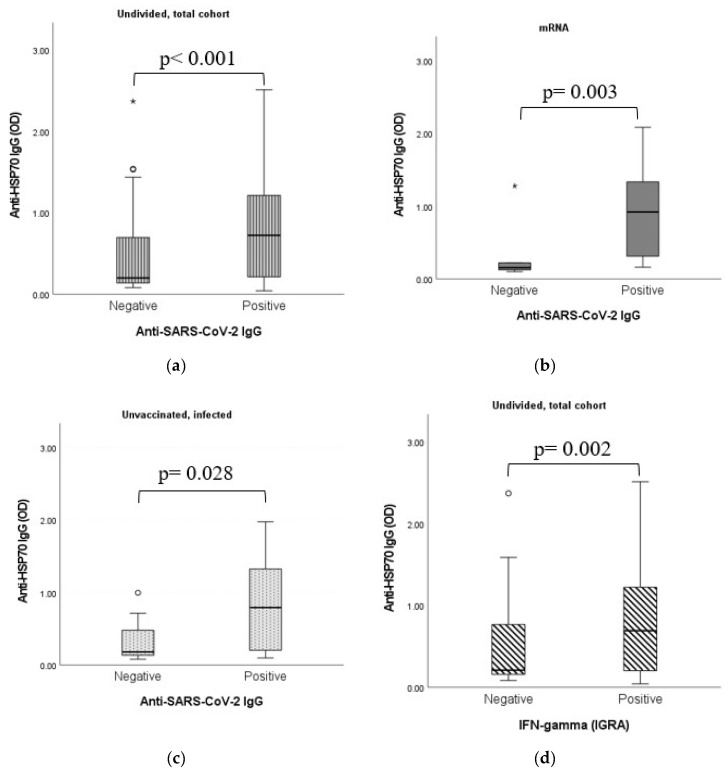
(**a**) Connection between anti-HSP70 IgG titers and anti-SARS-CoV-2 IgG results in the undivided, total sample cohort. (**b**) Connection between anti-HSP70 IgG titers and anti-SARS-CoV-2 IgG results in the mRNA vaccine group. (**c**) Connection between anti-HSP70 IgG titers and anti-SARS-CoV-2 IgG results in the unvaccinated, infected group. (**d**) Connection between anti-HSP70 IgG titers and IFN-γ (IGRA) results in the undivided, total sample cohort. Anti-SARS-CoV-2 IgG, IgA and IFN- γ (IGRA) qualitative (positive and negative) results were evaluated as per the manufacturer’s instructions. Anti-HSP70 IgG absorbance (OD) results were handled as continuous, non-normally distributed integers and the alterations of titers were considered. The sample numbers were as follows: (**a**) anti-SARS-CoV-2 IgG negative/positive = 48/136, (**b**) anti-SARS-CoV-2 IgG negative/positive = 12/15, (**c**) anti-SARS-CoV-2 IgG negative/positive = 12/15 and (**d**) IFN- γ (IGRA) negative/positive = 43/142. Total = 185.

As shown in Figure 7, statistically significant positive connections were found between the immunization-induced anti-SARS-CoV-2 humoral and cellular immune responses and the anti-HSP70 IgG levels.

### 2.8. Dynamic Adaptation of Anti-HSP70 IgG Antibodies

**Figure 8:**
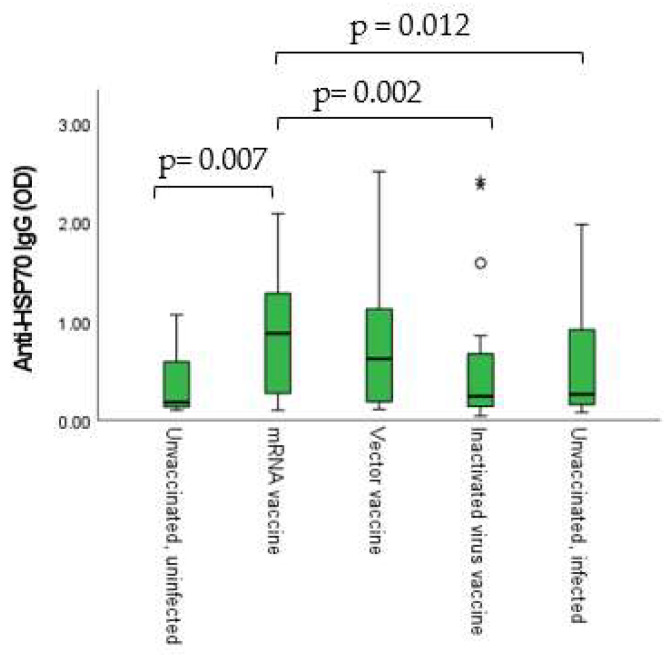
Vaccination group-based comparison of anti-HSP70 IgG titers. Anti-HSP70 IgG absorbance (OD) results were handled as continuous integers, non-normally distributed and the alterations of titers were considered. Total *n* =185.

Anti-HSP70 IgG levels showed statistically significant differences between the different vaccination groups. The difference was the most prominent between the inactivated virus vaccine and the mRNA vaccine groups (*p* = 0.002) (Figure 8).

### 2.9. Dynamic Adaptation of Anti-HSP60 IgG Antibodies 

**Figure 9:**
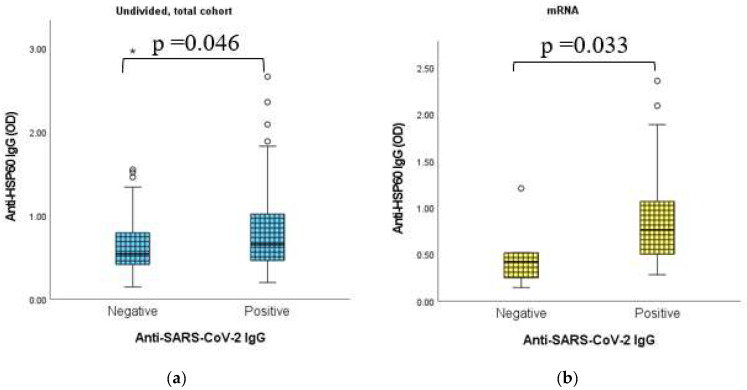
(**a**) Connection between anti-HSP60 IgG titers and anti-SARS-CoV-2 IgG results in the undivided, total sample cohort. (**b**) Connection between anti-HSP60 IgG titers and anti-SARS-CoV-2 IgG results in the mRNA vaccine group. Anti-SARS-CoV-2 IgG qualitative (positive and negative) results were evaluated as per the manufacturer’s instructions. Anti-HSP60 IgG absorbance (OD) results were handled as continuous, non-normally distributed integers and the alterations of titers were considered. The sample numbers were as follows: (**a**) undivided total cohort, anti-SARS-CoV-2 IgG negative/positive = 48/136 and (**b**) mRNA vaccine group, anti-SARS-CoV-2 IgG negative/positive = 6/63. Total = 185.

As shown in Figure 9, in the undivided total cohort, as well as in the mRNA vaccine group, statistically significant positive connection was found between the immunization-induced anti-SARS-CoV-2 IgG isotype of the humoral immune response and the anti-HSP70 IgG levels.

## 3. Discussion

### 3.1. Vaccine Efficacy

Vaccines designed to elicit protective and complex (cellular and humoral) immune responses remain the key for containing the COVID-19 pandemic caused by SARS-CoV-2. In particular, mRNA and vector vaccines have shown excellent efficacy when administered as two doses. Neutralizing responses are considered as a correlate of protection [50]. Lower antibody levels elicited by different COVID-19 vaccines are associated with breakthrough infections after vaccination, prompting consideration of booster doses or choosing a different type of vaccine [49]. Wild-type infection may enhance protection from vaccination—especially at the levels of IgA antibodies and cellular response—emphasizing the importance of a complex immune response (IR). In the first step, our objective was to examine the SARS-CoV-2 spike IgG, IgA antibodies, cellular response (using IGRA assay) and neutralizing antibody titers by comparing different vaccines (used in Hungary) to each other and to unvaccinated, infected (i.e., disease-experienced) individuals. 

Based on our results, it can be pronounced that recipients of mRNA and vector vaccines elicited the highest levels of IgG antibodies, while the unvaccinated, infected group (i.e., individuals who encountered only the wild-type virus, without pre-vaccination) and mRNA vaccine recipients showed the highest IgA levels. The highest cellular response (IGRA) was detected in the unvaccinated, infected group, followed by the mRNA and vector vaccine groups. However, it important to note that in Hungary, the inactivated virus vaccine has been administered mainly to individuals ≥ 60 years. This might have contributed to a suboptimal vaccine efficacy.

### 3.2. Potential Interference of Biological Therapies (Anti-TNFα and Anti-IL17) with Vaccine Efficacy

Because of the controversial literature data regarding the connection between immunomodulatory therapies and vaccination, we intended to find serological evidence for the potentially impaired anti-COVID-19 vaccine efficacy, using follow-up samples from patients treated with anti-IL-17A and anti-TNF-α therapies. The anti-SARS-CoV-2 IgA and IgG antibody levels of treated patients were compared with those of untreated healthy controls.

The human monoclonal antibody to IL-17 is a drug supplied with warnings about an increased risk of infections [49]. The risk of serious infections is unchanged or low over the short term; therefore, in the acute setting of COVID-19, the benefit of anti-IL-17 might outweigh the risks of infection [49]. According to current knowledge, IL-17 inhibition does not interfere with the capacity of patients to develop the expected responses to SARS-CoV-2 [42]. 

Regarding anti-TNFα therapy, clinical trials showed a moderate increase in upper respiratory infections (UTRIs) [45]. Though serum levels of TNFα have been noted to be elevated in patients with viral respiratory illnesses such as influenza, no significant elevation of TNFα was detected in the serum of patients with SARS, suggesting that the immune response to SARS may differ from that of other respiratory illnesses [42]. Anti-TNFα is a mainstream therapy in certain autoimmune conditions (e.g., IBD and psoriasis); however, it may be associated with increased susceptibility to infections and a lower vaccine response [44]. It has already been described that patients treated with anti-TNFα exhibited lower serologic responses one month and six months after vaccination (COVID-19 BNT162b2) compared to those not treated with anti-TNFα or to healthy controls (HCs) [44].

When comparing vaccine efficacy, we contrasted the results of biological therapy-treated patients (anti-TNFα or anti-IL17) at the two different time points (4 and 8 weeks post-vaccination) (Figure 3). We can conclude that the anti-SARS-CoV-2 IgA titers become visibly higher by the 8th week, although in statistical terms this proved to be only a trend (*p* = 0.091). At the level of anti-SARS-CoV-2 IgG titers, by the 8th week post-immunization, the visible increase in humoral response was also manifested in statistical terms (*p* = 0.024).

When comparing the anti-SARS-CoV-2 IgG and IgA levels of recipients of biological therapies with untreated healthy controls (Figure 4), it can be stated that the immunomodulatory therapies tend to interfere more negatively with the IgA isotype antibody production. In the case of patients receiving biological therapies, after the expectedly suboptimal IgA antibody levels at the 4th week post-immunization, even by the 8th week post-immunization the IgA levels remained lower compared to healthy individuals. Moreover, by the 8th week post-immunization, differences became even more prominent compared to healthy vector vaccine and healthy mRNA vaccine recipients (*p* = 0.037 and *p* = 0.040, respectively). 

Considering IgG production, the initial notable difference seen after 4 weeks diminished remarkably by the 8th week post-vaccination. At this latter time point, no significant differences were seen between treated patients and healthy controls of vector vaccines, although statistically significant differences were still detectable between patients receiving biological therapies and healthy recipients of mRNA vaccines (*p* = 0.002).

By the 8th week post-immunization, the difference in IgG titers between healthy recipients of vector vaccines and healthy recipients of mRNA vaccines had increased; it became significant also in statistical terms (*p* < 0.001). 

### 3.3. Vaccines (or Infections) and nAAbs

Infections or vaccines and autoimmunity are linked fields [33,34,35,51]. Recently, a link between COVID-19 and the development of autoimmunity has also been proposed [38,52]. It has also been suggested that SARS-CoV-2 causes the development of new-onset IgG autoantibodies. While pathological innate immune activation is well documented in severe diseases, the impact of autoantibodies on disease progression is less well defined [53]. A recent study concludes that COVID-19 patients exhibit dramatic increases in autoantibody reactivity compared to uninfected controls, with a high prevalence of autoantibodies against immunomodulatory proteins including cytokines, chemokines, complement components and cell surface proteins [53].

Indeed, our results suggest that in the case of elevated immunization-induced anti-SARS-CoV-2 antibody levels, the nAAb titers may be increased as well. This finding is in accordance with the literature data and our previous findings [54]. 

Although previously it has been hypothesized that IgM class nAAb titers are less prone to inducible fluctuations throughout life [3,55], according to recent findings [54] and our current results (Figure 5), and also supported by newer literature data [7], we suggest that this approach might be reviewed. A 2017 study performed on laboratory rats focused on the immunization-induced production of natural antibodies [7]. It was described that larger immunization-associated differences in natural antibody binding were seen with IgM antibodies compared to IgG antibodies. This fits with the dogma of immunoglobulin class switching, where the acute phase of primary antigen exposure is characterized by prominent IgM production [7], and it confirms the theory about the plasticity and the (restricted) capacity of adaptation of nAAbs.

HSP70 has already been described to work as a “double agent”, acting inside and outside the cell [56]. It has been found that Hsp70-derived epitopes interact with the immune cell components, consequently stimulating the humoral autoimmune response and production of the anti-HSP70 autoantibodies [56]. Considering the phenomenon of molecular mimicry, it has already been described that 17 human HSP proteins belonging to inter alia Hsp60, Hsp70 and Hsp90 chaperones share immunogenic epitopes (at least six amino acids) with SARS-CoV-2 proteins, as analyzed by the free Immune Epitope Database and Analysis Resource [57]. Evidently, the plasticity, being one of the most important features of the immune response, is also true in the case of HSPs [58]. Through molecular mimicry, the infection may cause the exposure of nonself-antigens to the immune system. The evolutionary conservation of heat shock proteins induces cross-reactivity with self-HSP antigens [58]. Considering all of these, it was expected that we would find connections between anti-HSP70 results and the immunization-induced immune response. In fact, a statistically significant positive connection was found between IgG isotype humoral antibody levels and anti-HSP70 results, as well as between IFN-γ release assays (IGRA) and anti-HSP70 results. Additionally, connections were observable between the different vaccination groups (vaccine types) and the anti-HSP70 IgG levels.

In the case of anticitrate synthase antibodies, we have earlier described that at the level of recognized epitopes, there is a possible link between the innate-like immunity and the adaptive autoimmune arm of the humoral immune system [11]. In a previous paper, we examined the potential association between adaptive antibodies (formed as a result of the childhood measles or MMR vaccine) and nAAbs, citrate synthase and the F4 fragment (anti-F4) of DNA topoisomerase I. We found significantly higher anti-CS IgG titers in the antimeasles IgG-sero-positive patient group (*p* = 0.011) compared to antimeasles IgG sero-negative individuals. The same trend was observable in the case of anti-F4 antibodies as well.

There is increasing evidence that in addition to disease-specific effects, vaccines against infectious diseases also exert nonspecific effects on the ability of the immune system to handle other pathogens [15]. Although epidemiological evidence for the nonspecific effects of vaccines is accumulating, the lack of biological feasibility has been an obstacle in the in-depth identification and study of these effects. Therefore, it is important to reveal those immunological mechanisms that may mediate nondisease-specific events [15].

## 4. Materials and Methods

### 4.1. Human Blood Samples

For the comparison of the natural autoantibody (nAAb) anti-citrate synthase (CS) (IgG, IgM) and the immunization (either vaccination or wild-type infection)-induced anti-COVID-19 antibodies (IgG), 255 anonymous serum samples were used (ethical license: 5726-PTE 2015, 5726/8216-PTE 2020). The subdivision of the totality of sera into smaller sample groups was set as follows: mRNA vaccine (Pfizer–BioNtech) group = 106, vector vaccines (AstraZeneca and Sputnik V) = 77, inactivated virus vaccine (Sinopharm) = 34, unvaccinated, uninfected individuals (i.e., individuals that have never encountered the virus or the vaccine, including in the form of a wild-type infection; they were used as negative controls) = 9, and unvaccinated, infected individuals (i.e., individuals that have encountered only the wild-type virus and without pre-vaccination; they were used as positive controls) = 29. (For the vaccine group nomenclature used for the figures, please see Supplementary Table S2).

For the comparison of the natural (auto)antibodies HSP60 and HSP70 (IgG, IgM) and the immunization (either vaccination or wild-type infection)-induced anti-COVID-19 antibodies (IgG), 185 anonymous serum samples were used (ethical license: 5726-PTE 2015, 5726/8216-PTE 2020). The subdivision of the totality of sera into smaller sample groups was set as follows: mRNA vaccine (Pfizer–BioNtech) = 69, vector vaccines (AstraZeneca and Sputnik V) = 56, inactivated virus vaccine (Sinopharm) = 24, unvaccinated, uninfected individuals (used as a negative control) = 9, and unvaccinated, infected individuals (used as a positive control) = 27. Blood draw was performed ~2 months after the second dose of the vaccine.

Statistical evaluation was performed using the Mann–Whitney U test, considering nAAb levels as ordinal, and non-normally distributed variables and immunization induced qualitative (positive and negative) results as the grouping parameters.

### 4.2. Human Serum Samples for the Comparison of Healthy Individuals versus Autoimmune Patients Receiving Biological Therapies

For the comparison of untreated, healthy individuals versus autoimmune patients (psoriasis and hidradenitis suppurativa) receiving biological therapeutic treatments (anti-IL-17A (Sekucinumab) and anti-TNFα (Adalimumab)), we received data from the Department of Dermatology, Venereology and Oncodermatology (Clinical Center of the University of Pécs, Hungary) belonging to 45 anonymous samples (ethical license: 8550-PTE 2020, 8550-PTE 2021).

The subdivision of samples was determined as follows: untreated, healthy individuals and recipients of the mRNA vaccine (Moderna and Pfizer–BioNtech) = 22, untreated, healthy individuals and recipients of vector vaccines (AstraZeneca and Sputnik V) = 16, and biological therapy-treated patients with mixed vaccination backgrounds (Pfizer–BioNtech, Moderna, Sputnik V, AstraZeneca and Sinopharm) = 7 (Supplementary Table S1). For the sample measurement and subsequent data analysis, three venipunctures were performed: time point zero was used as a negative control (on the day of immunization and before vaccine administration, when there were no detectable antibody levels; data is not shown), followed by time point one (4 weeks post-vaccination) and time point two (8 weeks post-vaccination) (Supplementary Table S2).

Anti-SARS-CoV-2 IgA and IgG antibody levels were analyzed in order to find potential differences between the following vaccine recipient groups: (a) psoriasis and hidradenitis suppurativa patients treated with the above detailed biological therapies and of mixed vaccination backgrounds, (b) healthy (untreated) individuals vaccinated with the mRNA vaccine and (c) healthy (untreated) individuals vaccinated with the vector vaccine. Antibody titers were compared at two time points, using serum samples from the 4th and 8th weeks post-vaccination. Statistical evaluation was performed using the paired t-test and Mann–Whitney U test.

### 4.3. Hsp60/Hsp70 IgG and IgM In-House ELISA Assays

Serum autoantibodies against human HSP60 (Abcam, Waltham, Boston, MA, USA) and human HSP70 (Abcam, Waltham, Boston, US) were evaluated using in-house developed ELISA. High-binding 96-well plates (Nunc maxisorp) were coated with the respective HSP at a concentration of 1 μg/mL in ELISA Coating Buffer (Bio-Rad, Hercules, CA, USA) at 4 °C overnight. The wells were blocked using an alternative combined blocking buffer (0.5% polyvinyl alcohol solution combined with bovine gelatin solution, at a ratio of 2:1) at room temperature (RT) for 2 h. After being washed with PBS + 0.05% Tween 20 (washing buffer; WB), sera were diluted (1:200 in WB) and incubated at RT for 1 h at 37 °C. The secondary antibodies were incubated at 37 °C for 40 min (horseradish peroxidase-conjugated antihuman IgG and IgM, polyclonal rabbit antihuman, (Agilent-Dako Santa Clara, CA, USA). TMB substrate solution (Sigma-Merck, Munich, Germany) was used to visualize the HRP enzymatic reaction, and the reaction was stopped by 1 M H_2_SO_4_. Reading was performed at λ = 450/620 nm using the BEP2000 Advanced automated system. Results are expressed in absorbance (OD) and in quantitative (standard curve-based) results. For data comparison, results were handled as continuous, non-normally distributed integers and the alterations of titers were considered.

### 4.4. Citrate Synthase (CS) IgG and IgM In-House ELISA Assays

Ninety-six well polystyrene plates (NUNC) were coated with CS from porcine heart (Sigma-Merck, Munich, Germany) in 0.1 M bicarbonate buffer, pH 9.6 [3]. Following this, the saturation of nonspecific binding sites with our alternative, combined blocking buffer (0.5% polyvinyl alcohol solution combined with bovine gelatin solution, at a ratio of 2:1) at room temperature (RT) for 2 h was performed. After being washed with PBS + 0.05% Tween 20 (washing buffer; WB), sera were diluted (1:100 in WB) and incubated 50 min at 37 °C. The secondary antibodies were incubated at 37 °C for 45 min (horseradish peroxidase-conjugated antihuman IgG and IgM, polyclonal rabbit antihuman (Agilent-Dako Santa Clara, CA, US). TMB substrate solution (Sigma-Merck, Munich, Germany) was used to visualize the HRP enzymatic reaction, and the reaction was stopped by 1 M H_2_SO_4_. Reading was performed at λ = 450/620 nm using the BEP2000 Advanced automated system. Results are expressed in absorbance (OD) and in quantitative (standard curve-based) results. For data comparison, results were handled as continuous, non-normally distributed integers and the alterations of titers were considered.

### 4.5. Neutralizing Antibody Measurement—Virus Neutralization ELISA (sVNT)

The NeutraLISA ELISA (EI 2606-9601-4 EUROIMMUN Medizinische Labordiagnostika AG) is a surrogate virus neutralization test (sVNT) for the determination of neutralizing antibodies that inhibit the binding of SARS-CoV-2 S1/RBD to ACE2 receptors. The SARS-CoV-2 NeutraLISA supports the evaluation of the individual immune response following SARS-CoV-2 infection or vaccination with S1-/RBD-based vaccines. If neutralizing antibodies are present in the sample, they compete with the receptor ACE2 for the binding sites of the SARS-CoV-2 S1/RBD proteins [59]. The ELISA assay was performed as per the manufacturer’s instructions. The test results obtained are given as percentages of inhibition (% IH). In the test, the ‘First WHO International Standard for anti-SARS-CoV-2 immunoglobulin (human) NIBSC code: 20/136’ is used as the reference material [60]. % IH < 20 negative; % IH ≥ 20 to 35 borderline; and % IH > 35 positive.

### 4.6. Interferon-γ ELISA

T cell-mediated interferon γ (IFN-γ) release after stimulation with SARS-CoV-2 spike peptides was assessed using Interferon-γ ELSIA by Euroimmun (EQ6841-3601 EUROIMMUN Medizinische Labordiagnostika AG). The IFN-γ release assay (IGRA) is used for the quantitative determination of the IFN-γ release by SARS-CoV-2-specific T cells. In this assay, heparinised whole blood is incubated in the three tubes [61]. The CoV-2 IGRA BLANK tube contains no activating components for immune cells; thus, no interferon-γ secretion is induced. The plasma thus obtained is used for individual background determination. The CoV-2 IGRA tube is coated with components of the S1 domain of the SARS-CoV-2 spike protein. The CoV-2 IGRA ‘STIM’ tube is coated with mitogen, causing an unspecific interferon-γ secretion. The plasma thus obtained is used to verify whether the sample contains immune cells in a sufficient quantity and with a sufficient ability to be activated [61]. The ELISA assay was performed as per the manufacturer’s instructions. The calibrator material of the test was adjusted to the international reference material ‘Non WHO Reference Material INTERFERON Γ (HUMAN, rDNA derived) NIBSC code: 87/586’ [62]. Results are expressed in international units per milliliter (mIU/mL). Result < 40 mIU/mL negative; result ≥ 40 to 50 mIU/mL borderline; and result > 50 mIU/mL positive.

### 4.7. Anti-SARS-CoV-2 ELISA (IgG, IgA)

SARS-CoV-2–specific IgG and IgA antibodies in serum were detected using anti–SARS-CoV-2 ELISA kits (EI 2606 A/G, both from EUROIMMUN). These kits allow for the specific detection of IgG and IgA isotype antibodies against SARS-CoV-2 using the S1 domain of the spike protein including the immunologically relevant receptor-binding domain (RBD)1. These indirect ELISAs provide a semi-quantitative in vitro determination of human antibodies against SARS-CoV-2 in serum. ELISA assays have been performed as per the manufacturer’s instructions. Results were expressed as a ratio of the extinction of the patient sample (or control) over the extinction of the calibrator. Ratio < 0.8 negative; ratio ≥ 0.8 to 1.1 borderline; and ratio > 1.1 positive.

### 4.8. Statistical Analysis

Data were analyzed using MS Excel for data assessment and SPSS for the Mann–Whitney U test; *p* values below 0.05 were considered statistically significant. Paired t-test was used in cases of low sample numbers.

## 5. Conclusions

Regarding vaccine efficacy, we found that Hungarian data are in accordance with international experiences: recipients of mRNA and vector vaccines had the highest humoral antiviral response in terms of IgG titers. The natural, wild-type infection (without pre-vaccination) and mRNA vaccines elicited the highest IgA levels. The highest induced IFN-γ (induced T cell response) levels were observed in unvaccinated, infected individuals, followed by mRNA and vector vaccine recipients.

Our current results may promote the acceptance of the role of nAAbs played in the individual varieties in immunological reactivity in response to vaccination. Nowadays, homeostatic nAbs and their target antigens still attract the most attention, although it is becoming more accepted that besides the formation of memory B cells and the ‘target’ antibodies, vaccination probably also has an unintended effect on the broadening and adaptation capacity of the nAb repertoire [7,8]. In this paper, we demonstrated the concomitance of vaccine (or infection)-induced antibodies and elevated levels of nAAbs with the ensuing positive statistical connections. Based on our current results, we also suggest that the earlier dogma regarding the relatively constant level of nAAbs throughout life—especially regarding the IgG isotype, which might resemble a transition between adaptive and innate immunity—might be reviewed. Based on our current and previous findings [54], it seems that these nAAbs, under certain circumstances, may be capable of showing a certain level of dynamic plasticity. Nevertheless, we would like to note that supplementing the current set of sera with pre-vaccination samples (obtained at time point zero, before the administration of the vaccine) and longer-term post-vaccination samples (e.g., 8 months after immunization), as well as a broader examination of the potentially expanded memory B cell functions (e.g., dynamic titer changes in different IgG subclasses, with special regard to IgG4) [63] might have contributed positively to the quality of the study.

Investigating the effect of biological therapies on the immunological response given to anti-SARS-CoV-2 vaccination, it can be concluded that the immunomodulatory therapies (anti-TNFα and anti-IL17) tended to interfere more negatively with the IgA isotopye of the humoral immune response, at both examined post-vaccination time points. Considering IgG isotype antibody production, by the 8th week post-vaccination no significant differences were seen anymore between treated patients and healthy recipients of vector vaccines, although statistically significant differences were still observable between patients receiving biological therapies and healthy recipients of mRNA vaccines. Our findings suggest differences between the specifically MALT-related and the systemic immune response to vaccines. Nonetheless, we would like to emphasize that for a more comprehensive conclusion, the herein presented modest sample numbers should be extended.

## Figures and Tables

**Table 1:** IgG and IgA isotype antibody sero-positivity ratios elicited by different vaccines.

Sero-Positivity Ratios	mRNA Vaccine	Vector Vaccine	Inactivated Virus Vaccine	Unvaccinated and Infected
Anti-SARS-CoV-2 IgG	95.88%	87.67%	69.23%	58.06%
Anti-SARS-CoV-2 IgA	65.38%	45.21%	22.58%	64.95%

**Table 2:** Cellular response (Anti-IFN-γ) and neutralizing antibody positivity ratios elicited by different vaccines.

Sero-Positivity Ratios	mRNA Vaccine	Vector Vaccine	Inactivated Virus Vaccine	Unvaccinated Infected
Anti-IFN-γ (IGRA) IgG	88.12%	87.01%	42.42%	93.10%
Neutralizing antibody (NeutraLisa IgA/G/M)	52.17%	53.95%	44.83%	44.83%

## Data Availability

Research data and investigation results are available upon request.

## Supplementary Materials

The following supporting information can be downloaded at https://www.mdpi.com/article/10.3390/ijms24076439/s1.

## Abbreviations

ACE2	Human angiotensin-converting enzyme 2
BCG	Bacillus calmette–guérin
CS	Citrate synthase
DTP	Diphtheria, tetanus and pertussis vaccine
HRP	Horseradish peroxidase
HSP	Heat shock protein
Ig	Immunoglobulin
IGRA	Interferon gamma release assay
IL	Interleukin
MMR	Measles, mumps and rubella vaccine
MS	Microsoft
MV	Measles vaccine
nAAb	Natural autoantibody
nAb	Natural antibody
NSE	Nonspecific side effect
PBS	Phosphate-buffered saline
PVA	Polyvinyl alcohol
RBD	Receptor-binding domain
RT	Room temperature
S1	Spike glycoprotein 1
SARS-CoV-2	Severe acute respiratory syndrome coronavirus 2
TMB	3,3′,5,5′-tetramethylbenzidine
TNF-α	Tumor necrosis factor alpha
URIs	Upper respiratory infections
WB	Washing buffer
