# Supplementary material for: Complexity of the Immune Response Elicited by Different COVID-19 Vaccines, in the Light of Natural Autoantibodies and Immunomodulatory Therapies
Source: Int J Mol Sci. 2023 Mar 29;24(7):6439. doi: 10.3390/ijms24076439 (PMC10094397; doi:10.3390/ijms24076439)
Supplement: Supplementary file 1 [file ijms-24-06439-s001.zip › ijms-2192352-supplementary.docx]

Supplementary Materials

**Table S1.** Anti-SARS-CoV-2 IgA and IgG antibody levels of psoriasis and hidradenitis suppurativa patients treated with biological therapies. Results were expressed as a ratio of the extinction of patient sample (or control) over the extinction of the calibrator. Ratio < 0.8-negative; ratio ≥ 0.8 to 1.1-borderline; ratio > 1.1–positive.

| Patient  Number | Age | Biological therapy | Vaccine | DAY 0 | | DAY 28 | | DAY 56 | |
| --- | --- | --- | --- | --- | --- | --- | --- | --- | --- |
|  |  |  |  | **IgA**  **(pos ≥1.1)** | **IgG**  **(pos ≥1.1)** | **IgA**  **(pos ≥1.1)** | **IgG**  **(pos ≥1.1)** | **IgA**  **(pos ≥1.1)** | **IgG**  **(pos ≥1.1)** |
| 1 | 57 | anti-IL17 | Pfizer-B. | 0,3 | 0,2 | 0,7 | 2,3 | 7,2 | >max |
| 2 | 71 | anti-IL17 | Pfizer-B. | 0,3 | 0,3 | >max | >max | 1,9 | >max |
| 3 | 62 | anti-IL17 | Sputnik V | 0,1 | 0,1 | 0,6 | 5,5 | 0,5 | 3,8 |
| 4 | 75 | anti-TNFα | Moderna | 0,3 | 0,2 | 0,5 | 1,4 | 1,6 | 7,9 |
| 5 | 39 | anti-TNFα | Sinopharm | 0,5 | 0,3 | 0,5 | 0,3 | 0,5 | 0,3 |
| 6 | 45 | anti-TNFα | Astra-Z. | 0,3 | 0,6 | 0,7 | 0,8 | 0,4 | 0,3 |
| 7 | 46 | anti-TNFα | Pfizer-B. | 0,3 | 0,2 | 0,4 | 2,7 | 0,8 | 6,9 |

**Table S2.** Vaccine group nomenclature and explanations*.*

| Group Name  (Used for Figures as Labels of the X Axes) | Vaccine | Explanation | Role in the Study |
| --- | --- | --- | --- |
| Unvaccinated, uninfected | N.A. | Individuals that have never encountered the virus nor in form of vaccine, neither in form of wild-type infection | negative control |
| mRNA vaccine group | Pfizer-Biontech | N.A. | vaccination cluster |
| Vector vaccine group | AstraZeneca, Sputnik V | N.A. | vaccination cluster |
| Inactivated virus vaccine | Sinopharm | N.A. | vaccination cluster |
| Unvaccinated, infected | - | Individuals that have encountered only the wild-type virus via natural infection, without pre-vaccination | positive control |
